# The prevalence and risk factors of peripheral neuropathy among patients with type 2 diabetes mellitus; the case of Jordan

**DOI:** 10.1186/s13098-018-0309-6

**Published:** 2018-02-21

**Authors:** Nahla Khawaja, Jawad Abu-Shennar, Mohammed Saleh, Said S. Dahbour, Yousef S. Khader, Kamel M. Ajlouni

**Affiliations:** 1 National Center (Institute) for Diabetes, Endocrinology and Genetics (NCDEG), The University of Jordan, P.O Box 13165, Amman, 11942 Jordan; 2 The University of Jordan, P.O Box 13165, Amman, 11942 Jordan; 3 Jordan University of Science and Technology, P.O Box 22110, Irbid, Jordan

**Keywords:** Diabetic peripheral neuropathy, Jordan, Prevalence

## Abstract

**Background:**

Peripheral neuropathy is one of the most common microvascular complication of diabetes mellitus. This study is conducted to determine the prevalence of diabetic peripheral neuropathy (DPN) and its associated factors among patients with type 2 diabetes mellitus in Jordan.

**Methods:**

A cross-sectional study was conducted at the National Center for Diabetes, Endocrinology and Genetics, Jordan. A total of 1003 patients with type 2 diabetes were recruited. Data were collected from participants during a face-to-face structured interview. DPN was assessed using the translated version of Michigan Neuropathy Screening Instrument (MNSI).

**Results:**

The overall prevalence of DPN based on MNSI was 39.5%. The most frequently reported symptoms were numbness (32.3%) and pain with walking (29.7%), while the least reported symptoms were the history of amputation (1.3%) and loss of sensation in legs/feet while walking (3.8%). Logistic regression analysis revealed that unemployment, cardiovascular disease, dyslipidemia, diabetic retinopathy and long standing DM (diabetes of ≥ 5 years) were significantly associated with DPN.

**Conclusion:**

Peripheral Neuropathy is highly prevalent among Jordanian patients with type 2 diabetes mellitus. DPN was significantly associated with duration of DM, dyslipidemia, diabetic retinopathy, cardiovascular disease, and unemployment. Early detection and appropriate intervention are mandatory among high-risk groups.

## Background

Diabetes mellitus (DM) is a devastating metabolic disorder that places an economic burden for every country around the world with the global increasing trend. As a cost of urbanization, the overall status of diabetes according to IDF estimates in 2017 showed that there are now 425 million adults with diabetes and 352 million adults with impaired glucose tolerance worldwide [1]. Jordan already has a high prevalence of DM and impaired fasting glucose approaching 24.9% in the year 2008 [2]. The age- adjusted prevalence rates in Saudi Arabia, Kuwait, Qatar and Jordan was 17.7, 15.8, 16.5 and 11.8%, respectively [1]. A great rise in diabetes prevalence is expected in Jordan due to alarming rates of obesity, economic growth, nutrition transition and a particular challenge of a large proportion of the resident population that consists of refugees and migrants from nearby countries including Syria and Iraq [1–3]. The overall prevalence of overweight and obesity among adult Jordanian was 31.4 and 51.7%, respectively [3]. According to WHO estimates, the highest levels of overweight and obesity from 16 countries in the Eastern Mediterranean Region were in Egypt, Bahrain, Jordan, Kuwait, Saudi Arabia and United Arab Emirates [4]. The prevalence of overweight and obesity in these countries ranged from 69 to 77% in men and 74 to 86% in women [4].

Neuropathy is the most common microvascular complications among diabetics that can involve peripheral, central and/or autonomic nervous systems [5–7]. It can also develop at earlier stages of dysglycemia as in the pre-diabetic phase [5, 7]. Peripheral neuropathy (PN) is the predominant variety in patients with diabetes whether type 1 or type 2. It manifests as distal symmetrical polyneuropathy (DSPN), also known as diabetic peripheral neuropathy (DPN), causing nerve damage in the extremities particularly the feet, in addition to radiculopathy and mononeuropathy [5, 6]. Clinically, DPN is defined as the presence of symptoms or signs of peripheral nerve dysfunction in people with diabetes after other possible causes have been excluded [8]. This definition reflects that the causality of neuropathy in diabetic patients is related to various factors in addition to hyperglycemia. However, the symptoms are not a reliable indicator for the presence of neuropathy in the disease course, since up to 50% of patients with neuropathy are asymptomatic; therefore, they are prone to insensate foot complications [9, 10]. Even in symptomatic patients, the spectrum of clinical manifestations is broad because of the complex anatomy of the peripheral, central and autonomic nervous systems.

DPN increases the risk of the foot infection, ulcers and non-traumatic amputations, eventually causing long-term disability [9, 11, 12]. The lifetime risk for the development of foot ulcers in patients with type 2 diabetes mellitus (T2DM) is 15–25% [9, 11]. DPN is one of three main risk factors for the occurrence of falls among patients with diabetes, along with vestibular dysfunction and diabetic retinopathy. In fact, the risk of falls is 2–3 times more likely in patients with DPN [13]. Overall, DPN has a significant impact on the quality of life, work productivity and health care resources utilization due to prolonged hospitalization [6, 14]. So far, the mainstay of treatment for DPN is pain management, while glycemic control is the only disease modifying therapy [6]. Therefore, peripheral neuropathy is one of the cardinal aspects when counseling diabetic patients and early detection in primary care setting is necessary to initiate the appropriate interventions in order to decrease disability, limb loss and improve the quality of life.

This study was conducted to determine the prevalence of DPN among patients with T2DM attending National Center for Diabetes, Endocrinology and Genetics (NCDEG) in Jordan and identify predictors of DPN.

## Methods

This study was cross-sectional in design conducted among adult patients (≥ 18 years) with T2DM who had regular follow up visits at the NCDEG for at least 6 months. Adult patients who underwent amputation of the whole foot, below knee and above knee amputations were excluded from the study. This study was conducted at NCDEG in Amman city, Jordan. Jordan is a small lower-middle income country with limited natural resources. Its surface area is about 89,300 square km, of which only 7.8% is arable land. Its population is about 9.8 million of whom 90.3% live in urban areas [15]. Around 629,245 of Syrians’ refugees are currently living in Jordan. Jordan’s health care system has improved dramatically over the last two decades and is ranked as one of the best systems in the Middle East. It is a complex amalgam of three major sectors: public, private, and non for profit organizations. The NCDEG is a national and specialized referral outpatient center that deals with cases of DM from all over Jordan and mainly serving a population of 4.1 million in Amman City. The NCDEG provides its services to the majority of patients with DM in Amman city, including those with health insurance. More than 70% of Jordanians had medical insurance. In 2017, NCDEG served 116,226 patients. Usual care of patients involves regular follow up every 3 months with weight, height and blood pressure measurements, fundus and foot examinations in addition to glycated hemoglobin (HbA1c). Yearly evaluation of microvascular complication in addition to lipid profile is a routine practice in all cases of DM in our center.

The study was approved by the ethics committee at the NCDEG. Eligible patients gave written informed consent and the confidentiality of the information was assured. The questionnaire and clinical examination were pilot tested on 100 participants (not included in this study) to ensure feasibility of the study and clear understanding of the inquiries. A total of 1003 patients with T2DM were recruited from the general diabetes clinics during the period from November 2014 to February 2015.

The socio-demographic data were collected from participants using a face-to-face structured interview, which included age, gender, marital status, regular visits to the treating physician every 3 months, family history of DM, physical and working status. The patient was considered to have regular, not regular and no physical activity if the patient walked 30 min for 4–7 days, 1–3 days/week, and less than once weekly, respectively. Clinical data related to the duration of diabetes, medications, the presence of nephropathy, diabetic retinopathy, hypertension, dyslipidemia cardiovascular disease, body mass index (BMI), and HbA1c were collected from medical files. HbA1c is measured using a high-performance liquid chromatography (HPLC) method with Bio-Rad VARIANT™ II Turbo 2.0 and D 10 Analyzer (Bio-Rad Laboratories).

The diagnosis of hypertension and dyslipidemia at the NCDEG are based on American Diabetes Association (ADA) criteria [16]. Nephropathy was defined as the presence of persistent albuminuria at levels 30–299 and levels ≥ 300 mg/24 h urine collection sample [16]. Cardiovascular disease (CVD) was defined as a previous diagnosis of coronary artery disease (CAD) by angiography or ECG, and or current treatment for CAD. Diabetic retinopathy diagnosis was based on dilated fundus examination carried out by experienced ophthalmologists.

The Michigan Neuropathy Screening Instrument (MNSI) was used to evaluate the presence of DPN [17]. Michigan Neuropathy Screening Instrument (MNSI) is a well-known instrument used to assess peripheral neuropathy among patients with T2DM with a sensitivity of 80% and a specificity of 95% [18, 19]. MNSI is a validated simple noninvasive and inexpensive measurement tool that incorporates sensory and motor components of neuropathy. Since its development, it became imperatively important to diagnose DPN as early as possible in order to prevent amputations, disability and improve the quality of life [17]. The MNSI consists of two-steps: The first step of MNSI is the history questionnaire that assessed the presence of neuropathic symptoms. Translation of the history part of the MNSI to the Arabic language was done by the researcher and checked by three professionals in the field of neurology, endocrinology, and nursing (It was valid and reliable through pilot testing of the instrument that was carried out on 100 patients). This part consists of 15 items, (13) items assessed symptoms of DPN, item number (4) assessed peripheral vascular disease (PVD), and item number (10) assessed general asthenia. The score range from 0 to 13 points and a score that is ≥ 7 indicates the presence of neuropathic symptoms. The second step of the MNSI is the physical examination part that was carried by the researchers. It assessed the following five variables on each foot: inspection of each foot for deformities, dry skin, calluses or infections, the presence or absence of ulceration, examination of vibration sense by tuning fork 128 Hz, the presence or absence of the ankle reflex using hummer and using Semmes–Weinstein Monofilament (SWM) to test sensation of feet. The researchers were healthcare professionals, trained by a professor of neurology at a university hospital for proper physical examination of MNSI tool and supervised by an endocrinologist at the NCDEG. The patient is considered to have neuropathy if the score is equal or more than two points on a 10 points scale on the physical examination part of MNSI. DPN based on MNSI scores were present if the score is equal or more than seven in the history part or a score equal or more than two in the physical examination part or both.

Data were analyzed by using the Statistical Package for Social Sciences (SPSS, version 20). Data were described using mean values for continuous variables and proportions for categorical variables. The *t* test was used to compare the means and Chi square was used to test independent distribution of categorical variables where appropriate. Binary logistic regression was used to identify independent predictors of peripheral neuropathy after adjusting all other potential confounders. A p value less than 0.05 was considered as statistically significant in the analysis.

## Results

### Participants’ characteristics

The study included a total of 1003 type 2 diabetic patients aged between 31 and 88 years with a mean age of 59.76 years (SD = 9.82). Their socio-demographic and clinical characteristics are shown in Table 1. More than half of the participants were females, 34% were unemployed and 62.1% were physically inactive. The mean BMI of study participants were 32 kg/m^2^ (SD = 5.63). The mean duration of diabetes was 9.24 years, almost one-third of participants were having diabetes for more than 12 years and 42% were having controlled diabetes. Microvascular complications in the form of retinopathy and nephropathy were present in 19.5 and 9.4%, respectively. The majority of study subjects were having dyslipidemia and almost all patients with dyslipidemia were receiving statin therapy. Moreover, 38.2 and 85.7% were having cardiovascular disease and hypertension, respectively (Table 1).

**Table 1:** Socio-demographic, clinical and laboratory data of the study participants

Variables	n	(%)
Age group (year)		
< 50	132	13.2
50–69	692	69.0
≥ 70	179	17.8
Gender		
Male	480	47.9
Female	523	52.1
Marital status		
Single/divorced and widowed	128	12.8
Married	875	87.2
Working status		
Unemployed	344	34.3
Employed	280	27.9
Retired	379	37.8
Physical activity		
Regular	92	9.2
Not regular	288	28.7
No physical activity	623	62.1
Family history of diabetes	869	86.6
Regular visit to treating physician	917	91.4
Body mass index (BMI) (kg/m^2^)^a^		
Normal	85	8.5
Over weight	302	30.1
Obese	616	61.4
Duration of diabetes (year)		
< 5	333	33.2
5–11	356	35.5
≥ 12	314	31.3
Hypertension	860	85.7
Dyslipidemia	886	88.3
Retinopathy	196	19.5
Nephropathy	94	9.4
Cardiovascular disease	383	38.2
Type of treatment		
Insulin only	128	12.8
Oral hypoglycemia agents only	527	52.5
Oral hypoglycemia agents and insulin	348	34.7
Statin therapy	886	88.3
HbA1C (%)		
Controlled < 7%	422	42.1
Uncontrolled ≥ 7%	581	57.9

^a^Normal: 18.5–24.9, over weight: 25–29.9 and obese ≥ 30

### Prevalence of DPN

The overall prevalence of DPN among study participants based on MNSI was 39.5%. Based on MNSI assessment, 16.5 and 34.8% of study participants had a score of ≥ 7 in the history questionnaire and a score of ≥ 2 in the physical examination section of the MNSI, respectively. Of those with DPN (396 patients), 88.1% had a score of ≥ 7 in the history questionnaire of the MNSI and 41.7% had a score of ≥ 2 in the physical examination section of the MNSI.

The history questionnaire of the MNSI assessment showed that most of the participants had at least one symptom of the DPN. The most frequently reported symptoms in DPN patients were numbness and pain with walking which was present in 81.8 and 75.3% of study participants, respectively while, the least reported symptoms were history of one or more toes amputation and loss of sensation in legs/feet while walking which was present in 3.3 and 9.6% of patients, respectively (Table 2).

**Table 2:** Responses to Michigan neuropathy screening instrument questionnaire in patients with diabetic peripheral neuropathy (n = 396)

Symptom	N (%)
A: History questionnaire	
Did your legs/feet numb?	324 (81.8)
Do you have burning pain in your legs/feet?	294 (74.2)
Are your feet too sensitive to touch?	155 (39.1)
Do you have prickling feelings in your legs/feet?	242 (61.1)
Does it hurt when the bed covers touch your legs/feet?	99 (25)
Can you differentiate hot water from cold water in the tub/shower?	59 (14.9)
Have you had open sore on your foot?	85 (21.5)
Has your doctor ever told you that you have neuropathy?	199 (50.3)
Are your symptoms worse at night?	276 (69.7)
Do your legs/feet hurt when you walk?	298 (75.3)
Are you able to sense your legs/feet when you walk?	38 (9.6)
Is the skin on your legs/feet so dry that it cracks open?	78 (19.7)
Have you had an amputation?	13 (3.3)

Physical examination	Right foot N (%)	Left foot N (%)
B: Physical assessment		
Abnormal appearance of feet	293 (74)	293 (74)
Ulceration	45 (11.4)	44 (11.1)
Ankle reflexes abnormality	198 (50)	203 (51)
Vibration perception abnormality	262 (66)	262 (66)
Monofilament test abnormality	314 (79)	310 (78)

The prevalence of DPN for study participants using MNSI according to relevant socio-demographic, clinical and laboratory characteristics is shown in Table 3.

**Table 3:** Prevalence of DPN for patients with type 2 DM using MNSI according to relevant socio-demographic, clinical and laboratory characteristics

Variables	Neuropathy status		p value
	Without DPN n = 607 (100%)	With DPN n = 396 (100%)	
Gender			0.137
Male	201 (41.9)	279 (58.1)	
Female	195 (37.3)	328 (62.7)	
Age group (year): (mean ± SD)	(62.7 ± 9.2)	(57.8 ± 9.7)	< 0.001*
< 50	23 (17.4)	109 (82.6)	
50–69	269 (38.9)	423 (61.1)	
≥ 70	104 (58.1)	75 (41.9)	
Marital status			0.777
Single/divorced and widowed	52 (40.6)	76 (59.4)	
Married	344 (39.3)	531 (60.7)	
Working status			< 0.001*
Unemployed	175 (50.9)	169 (49.1)	
Employed	83 (29.6)	197 (70.4)	
Retired	138 (36.4)	241 (63.6)	
Physical activity			< 0.001*
Regular	29 (31.5)	63 (68.5)	
Not regular	72 (25)	216 (75)	
No physical activity	295 (47.4)	328 (52.6)	
Family history of diabetes			0.014*
Present	356 (41)	513 (59)	
Absent	40 (29.9)	94 (70.1)	
Regular visit to treating physicians			< 0.001*
Yes	342 (37.3)	575 (62.7)	
No	54 (62.8)	32 (37.2)	
Body mass index (BMI) (kg/m^2^): (mean ± SD)	(32.9 ± 6.0)	(31.4 ± 5.3)	0.052
Normal	32 (37.6)	53 (62.4)	
Over weight	103 (34.1)	199 (65.9)	
Obese	261 (42.4)	355 (57.6)	
Duration of diabetes (year): (mean ± SD)	(14.1 ± 7.4)	(6.0 ± 5.1)	< 0.001*
< 5	29 (8.7)	304 (91.3)	
5–11	139 (39)	217 (61)	
≥ 12	288 (72.6)	86 (27.4)	
Having hypertension			< 0.001*
SBP mmHg (mean ± SD)	(142.4 ± 18.1)	(138.3 ± 17.5)	
DBP mmHg (mean ± SD)	(77.0 ± 11.3)	(78.2 ± 10.1)	
Yes	374 (43.5)	486 (56.5)	
No	22 (15.4)	121 (84.6)	
Nephropathy			< 0.001*
Yes	65 (69.1)	29 (30.9)	
No	331 (36.4)	578 (63.6)	
Cardiovascular disease			< 0.001*
Yes	210 (54.8)	173 (45.2)	
No	186 (30)	434 (70)	
Dyslipidemia			< 0.001*
Yes	381 (43)	505 (57)	
No	15 (12.8)	102 (87.2)	
TC (mean ± SD)	(178.6 ± 54)	(170.9 ± 47.3)	0.016
LDL (mean ± SD)	(101.0 ± 30.1)	(102.1 ± 31.1)	< 0.001*
HDL (mean ± SD)	(39.5 ± 11.3)	(41.9 ± 11.5)	0.001
TG (mean ± SD)	(177.4 ± 81.7)	(160.2 ± 71.8)	0.558
Statin therapy	381 (96.2%)	505 (83.2%)	< 0.001*
Retinopathy			< 0.001*
Yes	132 (67.3)	64 (32.7)	
No	264 (32.7)	543 (67.3)	
Type of treatment			< 0.001*
Insulin only	77 (60.2)	51 (39.8)	
Oral hypoglycemia agents only	120 (22.8)	407 (77.2)	
Oral hypoglycemia agents and insulin	199 (57.2)	149 (42.8)	
HbA1C (%): (mean ± SD)	(8.0% ± 1.5)	(7.1% ± 1.3)	< 0.001*
Controlled < 7%	96 (22.7)	326 (77.3)	
Uncontrolled ≥ 7%	300 (51.6)	281 (48.4)	

* Indicates statistically significant variables using x^2^ test at α < 0.05 level

### Multivariate analysis of factors associated with DPN

Multiple logistic regression analysis showed that unemployment, cardiovascular disease, dyslipidemia, diabetic retinopathy and duration of diabetes were significantly associated with DPN. Patients receiving oral hypoglycemic agents and having not regular physical activity were less likely to have DPN (Table 4). The likelihood of DPN was higher among patients with diabetic retinopathy (OR = 2.26, 95% CI: 1.5–1.3, *p* < *0.001*). Compared to patients who were physically inactive, patients with not regular physical activity (patients walked 30 min for 1–3 days/week) were less likely to have DPN (OR = 0.515, *p* = *0.001*). Cardiovascular disease and dyslipidemia were significantly associated with increased odds for DPN (OR = 1.43 and 2.23, respectively). Patients maintained on oral hypoglycemic agents were less likely to have DPN (OR = 0.639, *p* = 0.016) than those receiving insulin therapy in addition to oral hypoglycemic agents. Diabetes duration was the strongest predictor for DPN; compared to patients with T2DM for less than 5 years, those with diabetes duration for 5–11 years (OR = 5.25) and ≥ 12 years (OR = 16.98) were more likely to have DPN *(p* < *0.001).*

**Table 4:** Multivariate analysis of factors associated with DPN according to MNSI in patients with type 2 DM (n = 1003)

Variables	OR	95% confidence interval lower–upper	p value
Working status			
Retired	1		
Unemployed	1.86	1.27–2.73	0.001
Employed	1.4	0.92–2.14	0.112
Physical activity			
No physical activity	1		
Not-regular	0.515	0.35–0.75	0.001
Regular	0.712	0.40–1.25	0.239
Cardiovascular disease			
No	1		
Yes	1.43	1.02–2.01	0.037
Retinopathy			
No	1		
Yes	2.26	1.17–4.24	< 0.001
Dyslipidemia			
No	1		
Yes	2.23	1.17–4.24	0.014
Type of treatment			
Oral hypoglycemia agents and insulin	1		
Insulin only	1.17	0.72–1.91	0.509
Oral hypoglycemia agents only	0.639	0.44–0.92	0.016
Duration of diabetes (year)			
< 5	1		
5–11	5.25	3.29–8.36	< 0.001
≥ 12	16.98	10.19–28.28	< 0.001
Hypertension			
No	1		
Yes	1.06	0.56–2.01	0.839
HbA1C (%)			
Controlled < 7%	1		
Uncontrolled ≥ 7%	1.31	0.91–1.89	0.144
Body mass index (BMI) (kg/m^2^)			
Normal	1		
Over weight	0.776	0.42–1.43	0.418
Obese	0.816	0.45–1.46	0.493

1 Reference group

## Discussion

The current study intended to determine the prevalence of DPN and its correlates in patients with T2DM. The overall prevalence of DPN was 39.5%. There was no difference according to gender. Based on patients’ responses to MNSI: 41.9% of males were having DPN and 37.3% of females were having DPN (*p* = 0.137).

The results are consistent with several recent studies from Middle East countries in which the prevalence of DPN was 45, 31.9, 25.6 and 29.2% in Saudi Arabia, Iran, United Arab Emirates (UAE) and India, respectively [20–23] (shown in Table 5). Elrefai et al. and Al-Sarihin et al. conducted two previous studies in Jordan to assess the prevalence of DPN [24, 25]. Among 229 patients with diabetic foot disease, the prevalence of DPN was 89% [24]. The prevalence of DPN among 202 patients with type 1 and type 2 diabetes mellitus at tertiary referral hospital was 54.4% [25]. The differences in the reported prevalence rates in Jordan studies could be related to the differences in the study populations that included patients with type 1 diabetes mellitus and complicated patients with diabetic foot [24].

**Table 5:** The reported prevalence of DPN in various selected studies with their diagnostic criteria

Study	Country	Participants no. and type of DM	Neuropathy diagnostic tool	DPN prevalence %
Current study (2016)	Jordan	1003 Type 2	MNSI	39.5%
Elrefai et al. [24]	Jordan	229 patients with diabetic foot	One type of neuropathy: (presence of pain, abnormal ankle reflex, abnormal quantitative sensory testing)	89%
Al Sarihin et al. [25]	Jordan	86 Type 1 116 Type 2	MNSI	58.1% 51.7%
Al Mahroos et al. [35]	Bahrain	1477 Type 2	At least two abnormal tests: Neuropathy Symptom Score (NSS) Vibration Perception Threshold (VPT) Neuropathy Disability Score (NDS)	36.6%
Al Geffari et al. [20]	Saudi Arabia	242 Type 2	Abnormal two combined tests: (MNSI, Monofilament, vibration sensation, ankle reflex)	38.79%
Al-Kaabi et al. [22]	United Arab Emirates	394 Type 2	MNSI	25.6%
Pop-Busui et al. [26]	USA	2368 Type2 With CAD	MNSI	51%
Liu et al. [27]	China	1197 Type 2	Pressure sensation abnormality using 10-g monofilament and impaired vibration perception using tunning fork	17%
Tabatabaei-Malazy et al. [21]	Iran	124 patients	UK questionnaire MNSI Neuropathy Disability Score (NDS) Semmes Weinstein Monofilament (SWM)	54% 31.9% 38.1% 31.7%
Won et al. [29]	Korea	4000 Type 2	MNSI and 10-g monofilament	33.5%
Katulanda et al. [28]	Sri Lanka	528 Type2 191 New DM	Diabetic-Neuropathy-Symptom score (DNS)	DNS score: 48.1%
		337 known	Toronto-Clinical-Scoring-System (TCSS) and DNS	TCSS: 24%
Bansal et al. [23]	India	1637 new Type 2	One abnormal test: (10-g monofilament, pinprick sensation and ankle reflex)	9.2%
		396 known Type 2		33.7%
Kostev et al. [51]	Germany	New Type 2 45,633	Physician diagnosis or	5.7%
	UK	14,205	ICD code (E11.4)	2.4%

Pop-Busui et al. reported a higher prevalence among type 2 diabetic patients with angiographically documented CAD, which was 51% [26]. While the prevalence of DPN among Chinese patients newly diagnosed to have diabetes without cerebrovascular disease and foot ulcers using monofilament and tuning fork was 17% [27]. The discrepancy could be related to the inter subject variability in signs and symptoms of DPN and the differences in the sample selected and methods used to screen DPN [5].

Our data revealed that the likelihood of DPN was higher among patients with diabetic retinopathy (OR = 2.26, 95% CI: 1.5–1.3, *p* < *0.001*). Previous studies clearly demonstrated that diabetic patients having other microvascular and macrovascular complications were more likely to have DPN [22, 28–30]. This finding can be attributed to common pathogenic mechanisms as the toxic effect of hyperglycemia in the form of increasing thickness of endo-neural micro-vessels, accumulation of advanced glycation end products (AGEs), activation of the polyol pathway and oxidative stress [5, 31]. In the same context, our data showed that the odds ratio for DPN in patients with cardiovascular disease was 1.43 (CI: 1.02–2.01, *p* = 0.037). DPN is a known independent predictor of all and diabetes related mortality, and coronary artery disease is the leading cause of mortality in patients with DPN [32, 33]. Moreover, the risk of diabetic neuropathy was doubled in patients with cardiovascular disease independent of cardiovascular risk factors [30].

Among our study participants, the duration of diabetes was the strongest correlate of DPN (OR = 16.98, 95% CI: 10.19–28.28, *p* < *0.001*) for those who were having diabetes for ≥ 12 years and (OR = 5.25, 95% CI: 3.29–8.36, *p* < *0.001*) for those who were having diabetes for 5–11 years. This strong positive correlation was clearly demonstrated in several previous studies [22, 23, 25–30, 34].

In agreement with other reports, our data showed that patients with dyslipidemia were 2.23 times more likely to have DPN [28–30, 34, 35]. Current evidence supports the theory that metabolic syndrome and obesity are risk factors for DPN. The proposed mechanisms for nerve damage include extracellular protein glycation, fat deposition, oxidative stress, mitochondrial dysfunction and activation of counter-regulatory signaling pathways leading to chronic metabolic inflammation [36, 37]. Moreover, elevated triglycerides may serve as a blood marker for impairment of Schwann cell lipid metabolism and the pathological changes in the myelin structure of the nerve in cases of DPN [38]. Although no strong evidence for clear association between statin therapy and neuropathy is existing, still statin use could be another confounder for DPN among our study participants [36, 39, 40]. Statins usually interfere with cholesterol synthesis through inhibition of HMG-CoA reductase, which may alter nerve membrane function. Also, co-inhibit the synthesis of the key mitochondrial respiratory chain enzyme, ubiquinone, which may disturb neuron energy utilization and thereby induce neuropathy [40].

Our data showed that in comparison to patients who were physically inactive, those with not regular physical activity were less likely to have DPN (OR = 0.515, 95%CI: 0.35–0.75, *p* = 0.001). Contrary to our finding, physical inactivity was not a significant predictor of DPN in patients with DM in Sri Lanka [28]. This finding highlights the importance of physical activity in the prevention of DPN and adds more evidence to already existing data that links the lack of physical activity with DPN [22, 41, 42]. Physical activity will influence some of the underlying mechanisms of DPN in the form of microvascular dilatation, attenuation of oxidative stress and release of neurotrophic mediators in addition to its well-established role as inflammatory modulator and insulin sensitizer [41–43].

Although the prevalence of microvascular diabetic complications is strongly related to the degree of glycemic control, multivariate logistic regression analysis was unable to elicit an association between DPN and HbA1c level in the current study. Several studies evaluated the effect of glycemic control on neuropathy among patients with type 1, type 2 DM. Intensive glycemic control significantly reduced the development of both confirmed and definitive clinical neuropathy, and the benefits of prior intensive glycemic control for neuropathy were sustained in patients with type 1 DM [44–46]. While in type 2 diabetes this effect was less conclusive [47, 48]. The development of diabetic neuropathy is associated with a number of modifiable and non-modifiable risk factors as hypertension, dyslipidemia, insulin resistance, obesity, cigarette smoking and alcohol consumption in addition to the degree of hyperglycemia [21–23, 25–30, 37, 49]. The lack of association between DPN and HbA1c in the current study could be explained by the confounding effect of duration of diabetes where the intensity of glycemic control is affected by the duration of diabetes [29]. Moreover, the anti-hyperglycemic medications could play a role in attenuation of the impact of glycemic control on the development and progression of diabetic neuropathy [29]. In the meantime, our finding does not mean that proper glycemic control has no influence on the development of chronic DM complications as neuropathy.

Another finding in our study is the trend towards DPN protection in patients using oral hypoglycemic agents (OHA) as opposed to those using insulin therapy (OR = 0.639, 95%CI: 0.44–0.29, *p* = 0.016). This finding might be explained by the confounding effect of duration of diabetes as those who were on OHA had shorter duration of diabetes. The mean duration of DM for patients on OHA and insulin was 5.8 and 13.5 years, respectively. Furthermore, patients using insulin are already having more severe and advanced form of type 2 DM. In this study, 48.3, 27.3, 14.9 and 58% of patients receiving insulin were having CVD, retinopathy, nephropathy and neuropathy, respectively compared to 29, 12.5, 4.4 and 22.8% in patients using OHA (*p* < *0.001*). Pop-Busui et al. demonstrated that the 4-year cumulative incidence rate of DPN was significantly lower in patients using insulin sensitizing therapy (metformin and thiazolidinediones) than those using insulin providing therapy (sulfonylurea and insulin) (*p* = 0.020) [50]. Also, Kestev et al. had shown that insulin use was one of the strongest risk factors for DPN among newly diagnosed diabetics in Germany and UK [51]. Metformin that was used by 85.4% of our study participants has anti-inflammatory and a direct neuroprotective effect via inhibition of oxidative stress related apoptosis in primary neurons [28, 52, 53]. Exogenous insulin use in type 2 DM might reflect an advanced stage in the natural history of diabetes and could be associated with DPN by exacerbation of obesity, fluid retention, hypertension and hyperlipidemia [28, 29, 51, 54].

Our study is a clinic-based with large sample size, so our results may be applicable to patients receiving care in the community. The use of sensitive, highly predictive and inexpensive screening tool as MNSI to determine the prevalence of DPN will enhance the validity of the current study results. However, MNSI will assess only a large-fiber neuropathy but will not adequately assess small-fiber neuropathy, an important component of DPN. Other limitations are a cross sectional design, which could not evaluate the long-term effects of risk factors on the development of DPN as glycemic control. Further, the lack of inquiry about smoking, alcohol status and the use of medications affecting peripheral neuropathy and causality determination for neuropathy as B12 and folic acid deficiency are another limiting factors that could lead to overestimation of DPN prevalence.

Our data highlighted the need for intensive programs targeting at early detection of DPN and prompt implementation of health education in patients with dyslipidemia, cardiovascular disease, retinopathy and patients with long-standing diabetes. Moreover, initial measures to prevent DPN in the form of implementation of lifestyle and behavioral changes such as healthy eating patterns and exercise should be adopted to prevent the development and delay the progression of such a debilitating complication. Nevertheless, we need to keep in mind that action to stop diabetes epidemic is the best solution for preventing its complications.

## Conclusion

Diabetic peripheral neuropathy is highly prevalent among Jordanian patients with T2DM. Early detection and appropriate intervention are mandatory among patients with long standing DM, dyslipidemia, diabetic retinopathy, cardiovascular disease, and unemployment.

## References

[1] International Diabetes Federation (IDF) 2017. IDF Diabetes Atlas 8th ed. http://www.diabetesatlas.org/resources/2017-atlas.html. Accessed 22 Jan 2018.

[2] Ajlouni K, Khader YS, Batieha A, Ajlouni H, El-Khateeb M (2008). An increase in prevalence of diabetes mellitus in Jordan over 10 years. J Diabetes Complicat.

[3] Khader Y, Batieha A, Ajlouni H, El-Khateeb M, Ajlouni K (2008). Obesity in Jordan: prevalence, associated factors, comorbidities, and change in prevalence over ten years. Metab Syndr Relat Disord..

[4] World Health Organization. http://www.who.int/topics/obesity/en/. Accessed 22 Jan 2018.

[5] Tesfaye S, Selvarajah D (2012). Advances in the epidemiology, pathogenesis and management of diabetic peripheral neuropathy. Diabetes Metab Res Rev.

[6] Callaghan BC, Cheng HT, Stables CL, Andrea L, Smith AL, Feldman EL (2012). Diabetic neuropathy: clinical manifestations and current treatments. Lancet Neurol..

[7] Papanas N, Ziegler D (2012). Prediabetic neuropathy: does it exist?. Curr Diabetes Rep..

[8] Boulton AJ, Gries FA, Jervell JA. Guidelines for the diagnosis and outpatient management of diabetic peripheral neuropathy. Diabet Med 1998;15:508–514. https://doi.org/10.1002/(SICI)1096-9136(199806)15:6<508::AID-DIA613>3.0.CO;2-L. 10.1002/(SICI)1096-9136(199806)15:6<508::AID-DIA613>3.0.CO;2-L 9632127

[9] Wu SC, Driver VR, Wrobel JS, Armstrong DG (2007). Foot ulcers in the diabetic patient, prevention and treatment. Vasc Health Risk Manag..

[10] Boulton AJ, Vinik AI, Arezzo JC, Feldman EL, Freeman R, Malik RA, Maser RE, Sosenko JM, Ziegler D (2005). Diabetic neuropathies: a statement by the American Diabetes Association. Diabetes Care.

[11] Palumbo PJ, Melton LJ (1995). Peripheral vascular disease and diabetes. Diabetes Am.

[12] Rathur HM, Boulton AJ (2007). The diabetic foot. Clin Dermatol.

[13] Agrawal Y, Carey JP, Della Santina CC, Schubert MC, Minor LB (2010). Diabetes, vestibular dysfunction, and falls: analyses from the National Health and Nutrition Examination Survey. Otol Neurotol.

[14] Niranjan Y, Santwani MA, Baghel MS (2012). Quality of life consequences in diabetic polyneuropathy. Glob J Res Med Plants Indig Med..

[15] Jordan statistical yearbook 2016. Department of Statistics.

[16] American Diabetes Association (2014). Standards of medical care in diabetes. Diabetes care.

[17] Scale DE. Michigan Diabetes Research and Training Center. Online. https://diabetesresearch.med.umich.edu/Tools_SurveyInstruments.php. Accessed 22 Jan 2018.

[18] Herman WH, Pop-Busui R, Braffett BH, Martin CL, Cleary PA, Albers JW, Feldman EL (2012). Use of the Michigan Neuropathy Screening Instrument as a measure of distal symmetrical peripheral neuropathy in type 1 diabetes: results from the Diabetes Control and Complications Trial/Epidemiology of Diabetes Interventions and Complications. Diabetes Med.

[19] Moghtaderi A, Bakhshipour A, Rashidi H (2006). Validation of Michigan neuropathy screening instrument for diabetic peripheral neuropathy. Clin Neurol Neurosurg.

[20] Al-Geffari M (2012). Comparison of different screening tests for diagnosis of diabetic peripheral neuropathy in Primary Health Care setting. Int J Health Sci..

[21] Tabatabaei-Malazy O, Mohajeri-Tehrani MR, Madani SP, Heshmat R, Larijani B (2011). The prevalence of diabetic peripheral neuropathy and related factors. Iran J Public Health..

[22] Al-Kaabi JM, Al-Maskari F, Zoubeidi T, Abdulle A, Shah SM, Cragg P, Afandi B, Souid A (2014). Prevalence and determinants of peripheral neuropathy in patients with type 2 diabetes attending a tertiary care center in the United Arab Emirates. J Diabetes Metab.

[23] Bansal D, Gudala K, Muthyala H, Esam HP, Nayakallu R, Bhansali A (2014). Prevalence and risk factors of development of peripheral diabetic neuropathy in type 2 diabetes mellitus in a tertiary care setting. J Diabetes Investig..

[24] Elrefai JM (2009). Prevalence of neuropathy in the diabetic foot. Neurosciences.

[25] Al-Sarihin K, Althwabia I, Khaled MB, Haddad F (2013). Prevalence of peripheral neuropathy among patients with diabetes mellitus at King Hussein Hospital, Amman, Jordan. RMJ.

[26] Pop-Busui R, Lu J, Lopes N, Jones TL (2009). Prevalence of diabetic peripheral neuropathy and relation to glycemic control therapies at baseline in the BARI 2D Cohort. J Peripher Nerv Syst.

[27] Liu F, Bao Y, Hu R, Zhang X, Li H, Zhu D, Li Y, Yan L, Li Y, Lu J, Li Q, Zhao Z, Ji Q, Jia W (2010). Screening and prevalence of peripheral neuropathy in type 2 diabetic outpatients: a randomized multicenter survey in 12 city hospitals of China. Diabetes Metab Res Rev.

[28] Katulanda P, Ranasinghe P, Jayawardena R, Constantine GR, Sheriff MH, Matthews DR (2012). The prevalence, patterns and predictors of diabetic peripheral neuropathy in a developing country. Diabetol Metab Syndr.

[29] Won JC, Kwon HS, Kim CH, Lee JH, Park TS, Ko KS, Cha BY (2012). Prevalence and clinical characteristics of diabetic peripheral neuropathy in hospital patients with type 2 diabetes in Korea. Diabet Med.

[30] Rosson GD, Dellon AL (2005). Vascular risk factors and diabetic neuropathy. N Engl J Med.

[31] Ziegler D, Ametov A, Barinov A, Dyck PJ, Gurieva I, Low PA, Munzel U, Yakhno N, Raz I, Novosadova M, Maus J, Samigullin R (2006). Oral treatment with α-lipoic acid improves symptomatic diabetic polyneuropathy the SYDNEY 2 trial. Diabetes Care.

[32] Coppini DV, Bowtell PA, Weng C, Young PJ, Sönksen PH (2000). Showing neuropathy is related to increased mortality in diabetic patients—a survival analysis using an accelerated failure time model. J Clin Epidemiol.

[33] Hsu WC, Chiu SH, Yen AF, Chen LS, Fann CY, Liao CS, Chen HH (2012). Somatic neuropathy is an independent predictor of all-and diabetes-related mortality in type 2 diabetic patients: a population-based 5-year follow-up study (KCIS No. 29). Eur J Neurol.

[34] Booya F, Bandarian F, Larijani B, Pajouhi M, Nooraei M, Lotfi J (2005). Potential risk factors for diabetic neuropathy: a case control study. BMC Neurol.

[35] Al-Mahroos F, Al-Roomi K (2007). Diabetic neuropathy, foot ulceration, peripheral vascular disease and potential risk factors among patients with diabetes in Bahrain: a nationwide primary care diabetes clinic-based study. Ann Saudi Med.

[36] Callaghan B, Feldman E (2013). The metabolic syndrome and neuropathy: therapeutic challenges and opportunities. Ann Neurol.

[37] Smith AG, Singleton JR (2013). Obesity and hyperlipidemia are risk factors for early diabetic neuropathy. J Diabetes Complicat.

[38] Thomas PK, Ward JD, Watkins PJ (1982). Diabetic neuropathy: complications of diabetes.

[39] Macedo AF, Taylor FC, Casas JP, Adler A, Prieto-Merino D, Ebrahim S (2014). Unintended effects of statins from observational studies in the general population: systematic review and meta-analysis. BMC Med.

[40] Phan T, McLeod JG, Pollard JD, Peiris O, Rohan A, Halpern JP (1995). Peripheral neuropathy associated with simvastatin. J Neurol Neurosurg Psychiatry.

[41] Kluding PM, Pasnoor M, Singh R, Jernigan S, Farmer K, Rucker J, Sharma N, Wright DE (2012). The effect of exercise on neuropathic symptoms, nerve function, and cutaneous innervation in people with diabetic peripheral neuropathy. J Diabetes Complicat.

[42] Paul D, Loprinzi A, Kathy K, Hager B, Pradeep Y, Ramulu C (2014). Physical activity, glycemic control, and diabetic peripheral neuropathy: a national sample. J Diabetes Complicat.

[43] Loprinzi PD, Ramulu PY (2013). Objectively measured physical activity and inflammatory markers among US adults with diabetes: implications for attenuating disease progression. Mayo Clin Proc.

[44] Diabetes Control and Complication Trial Research Group (1995). The effect of intensive diabetes therapy on the development and progression of neuropathy. Ann Intern Med.

[45] Writing Team for the Diabetes Control and Complications Trial/Epidemiology of Diabetes Interventions and Complications Research Group: Control and Complications Trial/Epidemiology of Diabetes Interventions and Complications Research Group (2003). Sustained effect of intensive treatment of type 1 diabetes mellitus on development and progression of diabetic nephropathy: the Epidemiology of Diabetes Interventions and Complications (EDIC) study. JAMA.

[46] Martin CL, Albers J, Herman WH, Cleary P, Waberski B, Greene DA, Stevens MJ, Feldman EL (2006). Neuropathy among the diabetes control and complications trial cohort 8 years after trial completion. Diabetes Care.

[47] Ang L, Jaiswal M, Martin C, Pop-Busui R (2014). Glucose control and diabetic neuropathy: lessons from recent large clinical trials. Curr Diab Rep.

[48] Dziemidok P, Szczesniak G, Kostrzewa-Zablocka E, Paprzycki P, Korzon-Burakowska A (2012). Current glycaemic control has no impact on the advancement of diabetic neuropathy. Ann Agric Environ Med.

[49] Xu F, Zhao LH, Su JB, Chen T, Wang XQ, Chen JF, Wu G, Jin Y, Wang XH (2014). The relationship between glycemic variability and diabetic peripheral neuropathy in type 2 diabetes with well-controlled HbA1c. Diabetol Metab Syndr.

[50] Pop-Busui R, Lu J, Brooks MM, Albert S, Althouse AD, Escobedo J, Green J, Palumbo P, Perkins BA, Whitehouse F, Jones TL (2013). Impact of glycemic control strategies on the progression of diabetic peripheral neuropathy in the Bypass Angioplasty Revascularization Investigation 2 Diabetes (BARI 2D) cohort. Diabetes Care.

[51] Kostev K, Jockwig A, Hallwachs A, Rathmann W (2014). Prevalence and risk factors of neuropathy in newly diagnosed type 2 diabetes in primary care practices: a retrospective database analysis in Germany and UK. Prim Care Diabetes.

[52] Detaille D, Guigas B, Chauvin C, Batandier C, Fontaine E, Wiernsperger N, Leverve X (2005). Metformin prevents high-glucose-induced endothelial cell death through a mitochondrial permeability transition-dependent process. Diabetes.

[53] Isoda K, Young JL, Zirlik A, MacFarlane LA, Tsuboi N, Gerdes N, Schönbeck U, Libby P (2006). Metformin inhibits proinflammatory responses and nuclear factor-κB in human vascular wall cells. Arterioscler Thromb Vasc Biol.

[54] Savage S, Estacio RO, Jeffers B, Schrier RW (1997). Increased complications in noninsulin-dependent diabetic patients treated with insulin versus oral hypoglycemic agents: a population study. Proc Assoc Am Physicians.

