# Correction to: The prevalence and risk factors of peripheral neuropathy among patients with type 2 diabetes mellitus; the case of Jordan

**DOI:** 10.1186/s13098-018-0336-3

**Published:** 2018-05-18

**Authors:** Nahla Khawaja, Jawad Abu-Shennar, Mohammed Saleh, Said S. Dahbour, Yousef S. Khader, Kamel M. Ajlouni

**Affiliations:** 1 National Center (Institute) for Diabetes, Endocrinology and Genetics (NCDEG), The University of Jordan, P.O. Box 13165, Amman, 11942 Jordan; 2 The University of Jordan, P.O. Box 13165, Amman, 11942 Jordan; 3 Jordan University of Science and Technology, P.O. Box 22110, Irbid, Jordan

## Correction to: Diabetol Metab Syndr (2018) 10:8 10.1186/s13098-018-0309-6

It has been highlighted that the original article [1], published 21 February 2018, contained some errors in Table 3. This correction article shows the correct version of Table 3 and the changes that were made.

**Table 3:** The prevalence of peripheral neuropathy among patients with type 2 diabetes mellitus in Jordan according to relevant socio-demographic, clinical and laboratory characteristics

Variables	Peripheral neuropathy		P value
	Yes n = 396	No n = 607	
Gender			0.137
Male	201 (41.9)	279 (58.1)	
Female	195 (37.3)	328 (62.7)	
Age (year)			< 0.001
< 50	23 (17.4)	109 (82.6)	
50–69	269 (38.9)	423 (61.1)	
≥ 70	104 (58.1)	75 (41.9)	
Marital status			0.777
Single/divorced/widowed	52 (40.6)	76 (59.4)	
Married	344 (39.3)	531 (60.7)	
Working status			< 0.001
Unemployed	175 (50.9)	169 (49.1)	
Employed	83 (29.6)	197 (70.4)	
Retired	138 (36.4)	241 (63.6)	
Physical activity			< 0.001
Regular	29 (31.5)	63 (68.5)	
Not regular	72 (25)	216 (75)	
No physical activity	295 (47.4)	328 (52.6)	
Family history of diabetes			0.014
Present	356 (41)	513(59)	
Absent	40 (29.9)	94 (70.1)	
Regular visit to treating physicians			< 0.001
Yes	342 (37.3)	575 (62.7)	
No	54 (62.8)	32 (37.2)	
Body mass index (kg/m^2^)			0.052
Normal	32 (37.6)	53 (62.4)	
Over weight	103 (34.1)	199 (65.9)	
Obese	261 (42.4)	355 (57.6)	
Duration of diabetes (year)			< 0.001
< 5	29 (8.7)	304 (91.3)	
5–11	139 (39)	217 (61)	
≥ 12	288 (72.6)	86 (27.4)	
Having hypertension			< 0.001
Yes	374 (43.5)	486 (56.5)	
No	22 (15.4)	121 (84.6)	
Nephropathy			< 0.001
Yes	65 (69.1)	29 (30.9)	
No	331 (36.4)	578 (63.6)	
Cardiovascular disease			< 0.001
Yes	210 (54.8)	173 (45.2)	
No	186 (30)	434 (70)	
Dyslipidemia			< 0.001
Yes	381 (43)	505 (57)	
No	15 (12.8)	102 (87.2)	
Retinopathy			< 0.001
Yes	132 (67.3)	64 (32.7)	
No	264 (32.7)	543 (67.3)	
Type of treatment			< 0.001
Insulin only	77 (60.2)	51 (39.8)	
Oral hypoglycemia agents only	120 (22.8)	407 (77.2)	
Oral hypoglycemia agents and insulin	199 (57.2)	149 (42.8)	
HbA1c (%)			< 0.001
Controlled < 7%	96 (22.7)	326 (77.3)	
Uncontrolled ≥ 7%	300 (51.6)	281 (48.4)	

**Changes:**

1. A labeling error in Table 3 was corrected (Neuropathy status was replaced by Peripheral neuropathy).
2. [Without DPN n = 607 (100%)] was replaced by (Yes n = 396).
3. [With DPN n = 396 (100%)] was replaced by (No n = 607).
4. [Age group (year): (mean ± SD)] was replaced by [Age (year)], and the mean age was removed from the table.
5. [Body mass index (BMI) (kg/m^2^): (mean ± SD)] was replaced by [Body mass index (kg/m^2^)] and the mean BMI was removed.
6. [Duration of diabetes (year): (mean ± SD)] was replaced by [Duration of diabetes (year)] and the mean duration was removed.
7. SBP mmHg (mean ± SD) DBP mmHg (mean ± SD) was removed.
8. All these were removed TC (mean ± SD), LDL (mean ± SD), HDL (mean ± SD).
9. [HbA1C (%): (mean ± SD)] was replaced by [HbA1c (%)], and the mean HbA1c was removed from the table.
